# Beneficial Effects of Small-Molecule Oligopeptides Isolated from Panax Ginseng C. A. Meyer on Cellular Fates in Oxidative Stress-Induced Damaged Human Umbilical Vein Endothelial Cells and PC-12

**DOI:** 10.3390/ijms25052906

**Published:** 2024-03-02

**Authors:** Na Zhu, Yong Li, Meihong Xu

**Affiliations:** 1 Department of Nutrition and Food Hygiene, School of Public Health, Peking University, Beijing 100191, China; 2 College of Public Health, Inner Mongolia Medical University, Hohhot 010059, China; 3 Beijing Key Laboratory of Toxicological Research and Risk Assessment for Food Safety, Peking University, Beijing 100191, China

**Keywords:** ginseng oligopeptides, cell fate, senescence, oxidative stress, inflammation, mitochondrial function

## Abstract

Cell fate instability is a crucial characteristic of aging and appears to contribute to various age-related pathologies. Exploring the connection between bioactive substances and cell fate stability may offer valuable insights into longevity. Therefore, the objective of this study was to investigate the potential beneficial effects of ginseng oligopeptides (GOPs) isolated from Panax ginseng C. A. Meyer at the cellular level. Disruption of homeostasis of human umbilical vein endothelial cells (HUVECs) and PC-12 was achieved by culturing them in the growth medium supplemented with 200 µM of H_2_O_2_, and 25, 50, and 100 µg/mL GOPs for 4 h. Then, they were cultured in a H_2_O_2_-free growth medium containing different concentration of GOPs. We found that GOP administration retards the oxidative stress-induced cell instability in HUVECs by increasing cell viability, inhibiting the cell cycle arrest, enhancing telomerase (TE) activity, suppressing oxidative stress and an inflammatory attack, and protecting mitochondrial function. Furthermore, we hypothesized that GOPs may promote mitochondrial biosynthesis by upregulating PGC-1α expression. Similarly, GOPs positively regulated cell stability in PC-12; notably, the protective effect of GOPs on PC-12 mainly occurred through the inhibition of autophagic cell death of neuronal cells, while the protective effect on mitochondria was weak. In conclusion, it is evident that GOPs demonstrate potential beneficial effects in maintaining cell fate stability, thereby potentially contributing to an enhanced health span and overall well-being.

## 1. Introduction

The topic of a healthy life expectancy has gained significant attention globally due to the aging population and the subsequent rise in the prevalence of degenerative and chronic non-communicable diseases among elderly individuals [1]. Cells are the fundamental building blocks of the human body. Cell fate instability is indeed a key feature of aging and appears to be a common denominator and fundamental cause of age-related processes [2,3,4]. As organisms age, cells experience alterations in their fate determination and regulatory mechanisms, leading to a loss of cellular homeostasis and function. This instability in cell fate can manifest in various ways, such as impaired cellular differentiation, increased cellular senescence, and altered cellular signaling pathways. Exploring the mechanisms underlying this instability can provide valuable insights into the aging process and may pave the way for interventions to promote healthier aging and prevent age-related diseases.

Panax ginseng C. A. Meyer, commonly known as ginseng, has a long history of use as both a food and a medicinal herb in many cultures worldwide [5]. Ginseng has been recognized for its potential health benefits, and with the advancements in pharmacology and phytochemistry, various functions and bioactive components of ginseng have been identified and studied [6]. For the most part, scientists attributed the efficacy of ginseng to ginsenosides, which are the major functional ingredients [7]. It has been linked to a variety of benefits, including antioxidant, anti-inflammatory, anti-cancer, neuroprotective, and immunomodulatory effects. However, less attention has been paid to the nutrition side of ginseng. It is worth noting that ginseng is a natural plant that contains a rich source of various nutrients and bioactive compounds, such as polysaccharides, peptides, flavonoids, and minerals. These components contribute to the overall nutritional profile of ginseng and may have additional health-promoting effects. Exploring the nutritional aspects of ginseng and gaining a better understanding of its potential contributions to human health from a nutritional perspective can provide valuable insights into its overall health benefits.

The nutritional benefits of the bioactive peptides derived from the ginseng protein deserve attention. The molecular weight of bioactive peptides falls within the range between that of proteins and amino acids. Based on their size and structural properties, bioactive peptides exhibit a wide range of pharmacological functions, encompassing antioxidative, anti-inflammatory, hypotensive, hypoglycemic, and sedative activities [8,9,10,11]. These functionalities are intricately embedded within their sequences and are unleashed during the process of digestion. Peptides have garnered significant attention owing to their ability to bridge the divide between small molecules and protein drugs, thereby amalgamating the inherent advantages of both entities. Compared to dietary proteins and amino acids, food-derived bioactive peptides possess notable qualities such as high safety, rapid absorption, non-toxicity, and strong bioactivity [12]. These peptides can mimic the actions of endogenous peptides and, in some cases, influence the levels of endogenous active peptides, thereby working in synergy with them. Previous studies have demonstrated that peptides with lower molecular weights have a greater propensity to engage with free radical reaction sites and effectively participate in the oxidation reaction sequence. Moreover, shorter peptides comprising fewer than eight amino acids showcase significant antioxidant properties [13]. As of late, extensive literature has documented the advantageous effects of bioactive peptides in the context of aging and aging-related diseases [10,14].

GOPs are oligopeptides with molecular weights below 1000 Da, forming the foundation for their antioxidant mechanisms. Research indicates that peptides rich in polar amino acids exhibit heightened antioxidant activity by virtue of side chain chelation, which hinders free radical oxidation [8]. In our study, GOPs demonstrated an approximate polar amino acid content of 71.90%, further substantiating their robust antioxidant prowess. Our previous investigations have confirmed the anti-oxidative [15], anti-fatigue [16], immunomodulatory [17], and pancreatic dysfunction-improving [18] effects of GOPs. Furthermore, it has been observed that GOPs effectively mitigated oxidative stress-induced senescence in NIH/3T3 cells, markedly enhancing mitochondrial function and biogenesis through the NAD^+^/SIRT1/PGC-1α pathway [19]. These attributes have spurred our interest in exploring the potential of GOPs to impede the progression of age-related ailments and extend the overall health span.

The free radical theory of aging suggests that the accumulation of oxidative damage, whether induced by external factors or originating internally, increases as organisms age. This oxidative damage is believed to play a significant role in the process of senescence or mortality [20]. Recent evidence indicates that reactive oxygen species (ROS) contribute to the activation of compensatory homeostatic responses. However, it should be noted that higher concentrations of ROS can exacerbate age-related damage [21]. The fate of cells subjected to oxidative stress-induced damage is influenced by various factors, including the severity of stress, cellular defense mechanisms, and the capacity for repair. Cells can either recover and resume normal function, undergo programmed cell death, enter a state of cellular senescence, or experience an impaired regenerative capacity. Thus, understanding and modulating these cell fate outcomes in the context of oxidative stress is crucial for developing strategies to mitigate tissue damage and promote cellular health.

Each cell possesses a unique structure and function, contributing to the overall complexity and functionality of the human body. The manifestations of senescence also vary among different tissue cells [22]. For example, neurons, as terminally differentiated cells, exhibit aging patterns specific to their nature. To further investigate the relationship between cellular senescence and a healthy lifespan, as well as age-related diseases, we selected two distinct cell types: human umbilical vein endothelial cells (HUVECs) and the rat PC-12 cell line. Therefore, the H_2_O_2_-induced model was established in vitro. In this study, the beneficial effects of GOPs on cell fate instability were assessed, and the possible mechanisms were also observed, for the first time.

## 2. Results

### 2.1. Effect of GOPs on Oxidative Stress-Induced Damaged Cells

**Figure 1:**
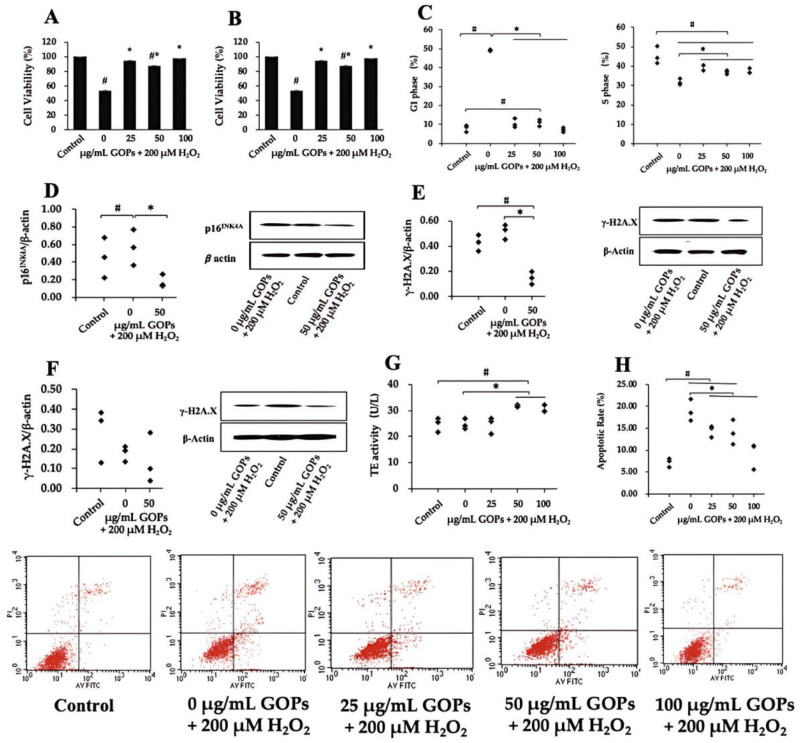
Effect of GOPs on the oxidative stress-induced damaged cells. (**A**) Cell viability evaluation of GOPs in HUVECs (*n* = 4 per group). (**B**) Cell viability evaluation of GOPs in PC-12 (*n* = 4 per group). (**C**) Effect of GOPs on the cell cycle distribution of HUVECs (*n* = 3 per group). (**D**) Effect of GOPs on the relative protein expression of p16^INK4A^ in HUVECs (*n* = 3 per group). (**E**) Effect of GOPs on DNA damage in HUVECs (*n* = 3 per group). (**F**) Effect of GOPs on DNA damage in PC-12 (*n* = 3 per group). (**G**) Effect of GOPs on telomerase (TE) activity (*n* = 3 per group). (**H**) Effect of GOPs on the cell apoptosis rate in PC-12 (*n* = 3 per group). Values represented the mean ± S.D. # *p* < 0.05 versus control group, * *p* < 0.05 versus H_2_O_2_-treated GOP-free group.

To assess whether GOPs have beneficial effects on cell fate instability, we tested several indicators. We found that GOP administration significantly increased the cell viability in oxidative stress-induced damaged HUVECs and PC-12 (Figure 1A,B), and the enhancement effect on PC-12 was more obvious (Figure 1B). GOP treatment also significantly inhibited the cell cycle arrest at the G1 phase and elevated the proportion of cells at the replication phase in HUVECs (Figure 1C). The expression of the cell cycle arrest-related protein P16^INK4A^ significantly reduced in GOP-supplemented HUVECs compared with the GOP-free group (Figure 1D). The protein expression of γ-H2A.X, the marker of DNA damage, significantly reduced in GOP-supplemented HUVECs compared with the control and the H_2_O_2_-treated GOP-free group (Figure 1E). On the other hand, GOP administration did not affect γ-H2A.X expression in PC-12 (Figure 1F). GOP administration significantly enhanced TE activity in GOP-supplemented HUVECs compared with the control and the H_2_O_2_-treated GOP-free group (Figure 1G). In addition, GOP administration significantly inhibited the oxidative stress-induced apoptotic rate in PC-12 (Figure 1H).

### 2.2. Effects of GOPs on Oxidative Stress Status

**Figure 2:**
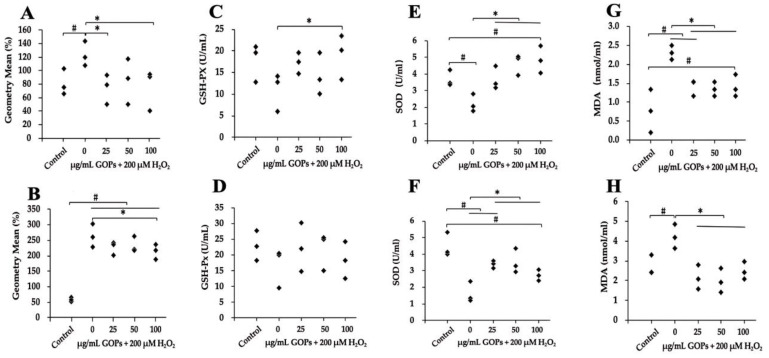
Effect of GOPs on the oxidative stress status of damaged cells. (**A**) Effect of GOPs on ROS production in HUVECs. (**B**) Effect of GOPs on ROS production in PC-12. (**C**) Effect of GOPs on GSH-Px activity in HUVECs. (**D**) Effect of GOPs on GSH-Px activity in PC-12. (**E**) Effect of GOPs on SOD activity in HUVECs. (**F**) Effect of GOPs on SOD activity in PC-12. (**G**) Effect of GOPs on MDA levels in HUVECs. (**H**) Effect of GOPs on MDA levels in PC-12. Values represent the mean ± S.D. (*n* = 3 per group). # *p* < 0.05 versus control group, * *p* < 0.05 versus H_2_O_2_-treated GOP-free group.

GOP administration (100 µg/mL) significantly decreased ROS production in both HUVECs and PC-12 compared with the H_2_O_2_-treated GOP-free group (Figure 2A,B). GOP administration (100 µg/mL) also significantly enhanced GSH-Px activity in HUVECs (Figure 2C), while no increase in PC-12 was detected compared with the H_2_O_2_-treated GOP-free group (Figure 2D). GOP administration significantly enhanced SOD activities and reduced MDA production in both HUVECs and PC-12 compared with the H_2_O_2_-treated GOP-free group (Figure 2E–H).

### 2.3. Effects of GOPs on Oxidative Stress-Induced Inflammation

**Figure 3:**
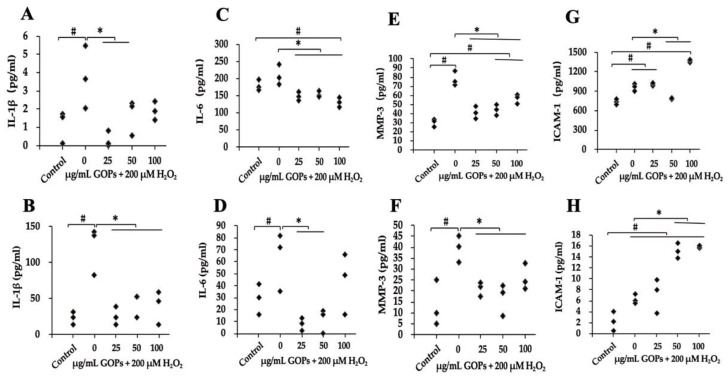
Effect of GOPs on oxidative stress-induced inflammation. (**A**) Effect of GOPs on IL-1β secretion in HUVECs. (**B**) Effect of GOPs on IL-1β secretion in PC-12. (**C**) Effect of GOPs on IL-6 secretion in HUVECs. (**D**) Effect of GOPs on IL-6 secretion in PC-12. (**E**) Effect of GOPs on MMP-3 secretion in HUVECs. (**F**) Effect of GOPs on MMP-3 secretion in PC-12. (**G**) Effect of GOPs on ICAM-1 secretion in HUVECs. (**H**) Effect of GOPs on ICAM-1 secretion in PC-12. Values represent the mean ± S.D. (*n* = 3 per group). # *p* < 0.05 versus control group, * *p* < 0.05 versus H_2_O_2_-treated GOP-free group.

GOP supplementation significantly inhibited the secretion of IL-1β, IL-6 and MMP-3 in both HUVECs and PC-12 compared with the H_2_O_2_-treated GOP-free group (Figure 3A–F). GOP administration (50 µg/mL) significantly reduced the secretion of ICAM-1 in HUVECs compared with the H_2_O_2_-treated GOP-free group, while a higher dose of GOP exposure significantly elevated the secretion of ICAM-1 in HUVECs compared with the H_2_O_2_-treated GOP-free group and control group (Figure 3G). In addition, 50 and 100 µg/mL of GOP supplementation significantly increased the production of ICAM-1 in PC-12 compared with the control and the H_2_O_2_-treated GOP-free group (Figure 3H).

### 2.4. Effects of GOPs on Mitochondrial Function

**Figure 4:**
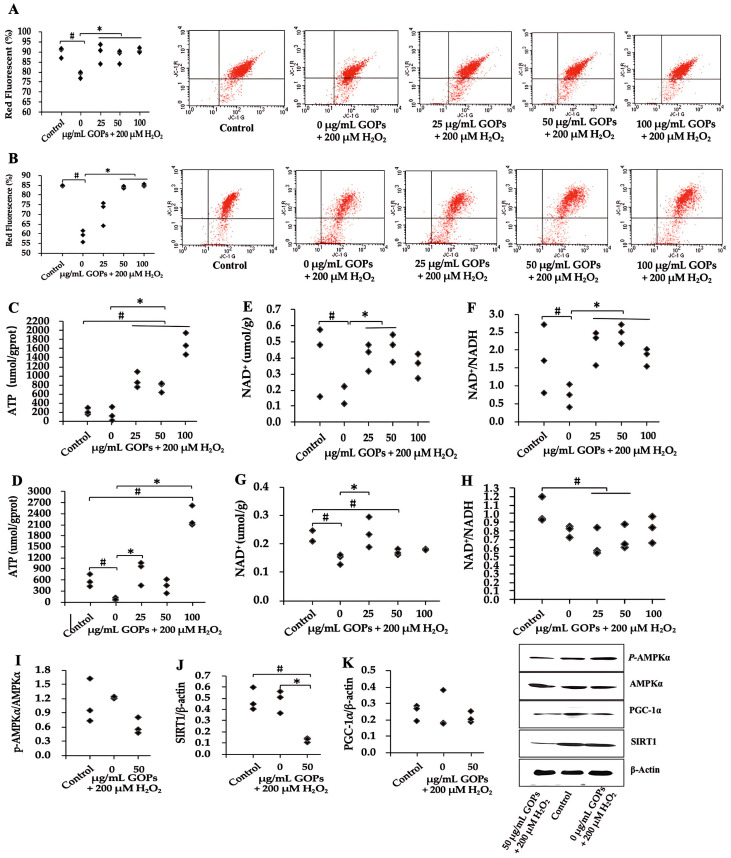
Effect of GOPs on mitochondrial function and biogenesis. (**A**) Effect of GOPs on MMP in HUVECs. (**B**) Effect of GOPs on MMP in PC-12. (**C**) Effect of GOPs on the ATP level in HUVECs. (**D**) Effect of GOPs on the ATP level in PC-12. (**E**) Effect of GOPs on NAD^+^ activity in HUVECs. (**F**) Effect of GOPs on the NAD^+^/NADH in HUVECs. (**G**) Effect of GOPs on the NAD^+^ activity in PC-12. (**H**) Effect of GOPs on the NAD^+^/NADH in PC-12. (**I**) Effect of GOPs on the relative protein expression of p-AMPK/AMPK in HUVECs. (**J**) Effect of GOPs on the relative protein expression of SIRT1 in HUVECs. (**K**) Effect of GOPs on the relative protein expression of PGC-1α in HUVECs. Values represent the mean ± S.D. (*n* = 3 per group). # *p* < 0.05 versus control group, * *p* < 0.05 versus H_2_O_2_-treated GOP-free group.

Compared with the H_2_O_2_-treated GOP-free group, GOP administration significantly enhanced the mitochondrial membrane potential (MMP) in both oxidative stress-induced damaged HUVECs and PC-12 (Figure 4A,B). There is a remarkable outcome that GOP supplementation strongly increased ATP production in both oxidative stress-induced damaged HUVECs and PC-12, increased by four or more times compared with the H_2_O_2_-treated GOP-free group (Figure 4C,D). GOP administration also significantly enhanced NAD^+^ activity and NAD^+^/NADH in oxidative stress-induced damaged HUVECs compared with the H_2_O_2_-treated GOP-free group (Figure 4E,F). Contrastingly, just 25 µg/mL GOP administration significantly enhanced NAD^+^ activity and significantly decreased NAD^+^/NADH in oxidative stress-induced damaged PC-12 compared with the H_2_O_2_-treated GOP-free group (Figure 4G,H). We further estimated the effect of GOPs on the mitochondrial biogenesis signaling pathway AMPK/NAD^+^/SIRT1/PGC-1α in oxidative stress-damaged HUVECs. Compared with the control and the H_2_O_2_-treated GOP-free group, GOP administration significantly reduced the protein expression of SIRT1 in HUVECs (Figure 4J). No significant differences were found between the groups on the protein expression of p-AMPK/AMPK and PGC-1α in damaged HUVECs (Figure 4I,K). We suggest that it cannot yet be confirmed that GOPs fail to up-regulate PGC-1α expression because GOPs significantly enhanced NAD^+^ activity, whereas p-AMPK/AMPK and SIRT1 expression tended to be drastically down-regulated or significantly down-regulated. It can be hypothesized that GOPs had no effect on AMPK and SIRT1 expression, resulting in a large depletion of AMPK and SIRT1 without replenishment during mitochondrial synthesis, whereas GOPs may have an up-regulatory effect on PGC-1α, and thus there was not a substantial reduction in PGC-1α during mitochondrial synthesis.

### 2.5. Effects of GOPs on Autophagy in Damaged PC-12

**Figure 5:**
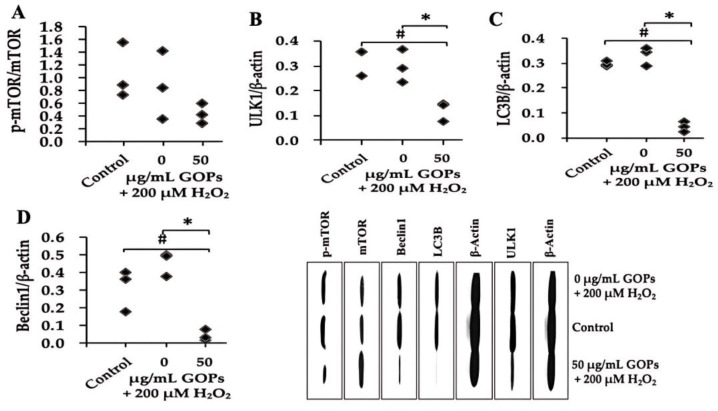
Effect of GOPs on autophagy in damaged PC-12. (**A**) Effect of GOPs on the relative protein expression of p-mTOR/mTOR in PC-12. (**B**) Effect of GOPs on the relative protein expression of ULK1 in PC-12. (**C**) Effect of GOPs on the relative protein expression of LC3B in PC-12. (**D**) Effect of GOPs on the relative protein expression of Beclin1 in PC-12. Values represent the mean ± S.D. (*n* = 3 per group). # *p* < 0.05 versus control group, * *p* < 0.05 versus H_2_O_2_-treated GOPs free group.

GOP administration did not statistically affect the relative protein expression of p-mTOR/mTOR compared with the control and the H_2_O_2_-treated GOP group in PC-12 (Figure 5A). GOP administration (50 µg/mL) significantly down-regulated the protein expression of ULK1, LC3B, and Beclin1compared with the control and the H_2_O_2_-treated GOP-free group (Figure 5B–D).

## 3. Discussion

Building upon previous studies conducted in our laboratory, we aimed to investigate the potential effects of GOPs on the health span. To explore the implications of GOPs on the aging process and elucidate their underlying mechanisms, a series of experiments were conducted using a biological model consisting of cultured HUVECs and PC12 cells. These cell choices serve specific research purposes: cardiovascular diseases are the leading cause of death worldwide. The aging process involves a series of anatomical and histological degenerative changes and the decline in physiological functions in the cardiovascular system. These changes play a significant role in the occurrence and development of cardiovascular diseases in the elderly. HUVECs are commonly utilized in studies related to cardiovascular diseases, angiogenesis, vascular permeability, and drug screening for therapies targeting the vascular system. On the other hand, life expectancy has significantly expanded the number of elderly individuals with neurodegenerative disorders, such as Parkinson’s disease and Alzheimer’s disease, as the major risk factor for developing these conditions is age. PC12 cells find frequent application in neurobiology research, exploring neurotoxicity, neurodegenerative diseases, and neuronal differentiation studies. Various factors are known to trigger premature senescence or cell death, independent of telomeric processes [23]. Among these factors, oxidative stress induced by ROS plays a significant role, making it a crucial focus in scientific research, including our study [24]. By utilizing an oxidative stress-induced model, we aimed to replicate the conditions observed in naturally senescent or dying cells. This model has been widely employed in scientific investigations to study the effects of oxidative stress on cellular aging and related processes [25,26,27]. Thus, in the current study, we adopted this approach to assess the impact of GOPs on cell fate in the context of oxidative stress-induced cell senescence or death.

We conducted a comprehensive assessment of several cell fate markers during the experiment. Our data demonstrated that GOPs effectively delayed senescence in HUVECs and cell death in PC-12 cells induced by oxidative stress. Senescent HUVECs exhibited an accelerated decline in cell viability and proliferation. It is known that irreparable DNA damage can induce senescence [28], and γ-H2A.X is considered a biomarker of DNA damage [29]. We observed a tendency for the 200 µM H_2_O_2_ treatment to accelerate DNA damage. We report that GOPs delayed oxidative stress-induced damage in HUVECs and PC-12 cells. GOPs effectively enhanced cell viability in HUVECs and PC-12. Similarly, the apoptosis level of PC-12 cells was significantly increased in H_2_O_2_-induced groups. Moreover, GOP treatment significantly promoted DNA synthesis at the S phase and inhibited the cell cycle arrest. Correspondingly, cell viability and apoptosis rates returned to normal levels. Further analysis of the data provided comprehensive evidence supporting the positive role of GOPs. Considering that cellular senescence is recognized as a hallmark of aging, therapeutic strategies targeting senescent cells may attenuate age-associated pathologies and extend the lifespan. Our data revealed the role of GOPs in delaying HUVEC senescence and suppressing PC-12 apoptosis, thus highlighting its potential application in the therapy of cardiovascular diseases (CVDs) and neurodegenerative diseases.

Recent studies have highlighted that senescence-associated secretory phenotype (SASP), a hallmark of cellular senescence, can contribute to age-related pathologies through paracrine and endocrine effects [30,31]. Consequently, suppressing SASP is a rational approach to retard senescence and attenuate age-related diseases. Our study demonstrates that GOPs possess potent anti-inflammatory properties in two kinds of cell lines, consistently reducing the levels of inflammatory cytokines such as IL-6 and IL-1β. This indicates that GOPs have the ability to suppress SASP. Moreover, GOPs play a crucial role in maintaining optimal immune responses, as supported by previous research indicating their anti-inflammatory effects [17]. Considering that oxidative stress exacerbates age-related damage, GOPs may alleviate oxidative damage and enhance antioxidant capacity, thereby retarding damage in cells. Our findings reveal that GOPs exhibit superior antioxidant properties through different mechanisms, which could be a significant strategy in combating cellular damage [32]. The proinflammatory transcription factor NF-κB is a major regulator of SASP, and GOP administration has been shown to suppress NF-κB activation, contributing to their anti-inflammatory effects. Similar observations have been made in previous in vivo experiments, where GOPs down-regulated NF-κB and inhibited cytokine secretion in mouse models with varying degrees of inflammation [15,18]. Additionally, GOPs’ anti-inflammatory properties may also be attributed to their DNA-protective effect, as genomic instability is a fundamental driver of SASP and NF-κB activation is initiated by the DNA damage response. The aging process is characterized by a complex interplay between various hallmarks, including the recently identified hallmarks of health [21,33]. Furthermore, we speculate that GOPs regulate the feedback loop between SASP, ROS production, and DNA damage responses [34], thereby combating senescence progression from multiple angles. Additionally, our previous studies have shown that GOPs inhibit the decline in antioxidant enzyme activity and the increase in lipid peroxidation products in aged animal models [23].

There is strong evidence indicating that oxidative stress plays a significant role in various age-related pathologies [35]. Mitigating oxidative damage is a crucial strategy for combating the aging process. Our study demonstrated that GOPs exhibit a moderate ability to scavenge free radicals, particularly at lower dosages. This finding can be attributed to the overwhelming levels of both exogenously supplied and endogenously generated reactive oxygen species (ROS), which surpass the capacity of natural antioxidant GOPs to neutralize them fully. However, further analysis revealed that GOPs significantly enhance the antioxidant enzyme system and tend to reduce the production of malondialdehyde (MDA) during the damage process in HUVECs and PC-12 cells [15,16,17]. These findings are consistent with our previous observations, which showed that GOPs effectively increase the activities of antioxidant enzymes such as superoxide dismutase (SOD) and glutathione peroxidase (GSH-px), while decreasing MDA levels in mice.

Our laboratory’s previous investigations have revealed the ability of GOPs to enhance mitochondrial function in mice skeletal muscles. This enhancement is achieved by augmenting mitochondrial DNA content and promoting the expression of NRF-1 and TFAM genes [16]. Mitochondria play a critical role in the aging process, as persistent mitochondrial dysfunction during aging increases the production of ROS, which subsequently exacerbates mitochondrial degradation and cellular damage [21]. Therefore, our research aimed to explore the impact of GOPs on mitochondrial activity. The outcomes of our study unveiled that GOP supplementation improved mitochondrial function, as demonstrated by an elevation in the mitochondrial membrane potential in HUVECs and PC-12 cells. These findings align with numerous investigations highlighting the potential of bioactive peptides derived from marine sources to repair or enhance mitochondrial function following exposure to external stressors like H_2_O_2_ and UV radiation [36,37,38]. Age-related mitochondrial dysfunction is frequently associated with an impaired turnover, characterized by reduced biogenesis and clearance of mitochondria [21]. The AMPK/NAD^+^/SIRT1/PGC-1α signaling pathway, a well-established pathway involved in mitochondrial biogenesis, is closely linked to the regulation of longevity [39]. In our study, GOP supplementation significantly elevated NAD^+^ levels and NAD^+^/NADH activity, while upregulating the protein expression of PGC-1α in vitro. Simultaneously, the relative protein expression of SIRT1 was lower in the H_2_O_2_-treated GOP-free group compared to the GOPs-treated group. As SIRT1 relies on NAD^+^ for its function, this observation can be attributed to NAD^+^ depletion in the H_2_O_2_-treated GOP-free group, resulting in a relatively higher SIRT1 expression due to the reduced SIRT1 consumption.

Additionally, we observed autophagic cell death in PC-12 cells. Autophagy is a cellular self-degradation process where a portion of the cytoplasm is enclosed within double-membrane or multi-membrane vesicles known as autophagosomes. These autophagosomes are subsequently transported to lysosomes for extensive degradation. The initiation phase of autophagy involves the formation of phagophores, which engulf cytoplasmic material, followed by elongation and fusion of phagophore membranes to enclose autophagosomes. The fusion of autophagosome outer membranes with lysosomes results in the formation of autolysosomes (also referred to as autophagolysosomes), where the luminal contents, including the inner membrane, undergo degradation. The breakdown products are released through permeases and circulate within the cell’s solute. Hence, autophagy is a mechanism by which non-nuclear cell components can be renewed, and cellular macromolecules can be mobilized to generate energy-rich compounds, thereby meeting the bioenergetic demands of cells during periods of external or internal resource depletion [40]. Autophagy plays a dual role in neuronal cells. On one hand, it serves as a major mechanism for clearing misfolded and abnormal proteins in neurodegenerative diseases. On the other hand, autophagy can activate cell death pathways, resulting in autophagic cell death [41]. mTOR is a negative regulator of autophagy in organisms. Under starvation conditions, mTOR complex 1 dissociates from its direct activator located in lysosomes, leading to mTOR inhibition. This inhibition contributes to starvation-induced autophagy by activating mTOR target proteins, such as Atg13, ULK1, and ULK2. When nutrient availability is sufficient, mTOR-associated lysosomes move toward the plasma membrane, which is crucial for mTOR activation and leads to a reduction in autophagosome formation [42]. In this study, when compared to the blank control group, the model control group exhibited a decreasing trend in mTOR expression, and ULK1, an autophagy-inducing protein, also showed a decreasing trend. However, autophagy-related proteins, Beclin1 and LC3B, displayed an increasing trend, suggesting that hydrogen peroxide intervention may promote autophagic cell death in neuronal cells. In comparison to the H_2_O_2_-treated GOP-free group, intervention with GOPs significantly inhibited autophagy-related proteins ULK1, Beclin1, and LC3B, indicating that GOPs may reduce neuronal autophagic cell death by suppressing the expression of autophagy-related proteins.

Indeed, our study provides evidence that GOPs effectively retard oxidative stress-induced damage in HUVECs and PC12 cells. The observed improvements include decreased expression of senescence markers, enhanced cell viability, increased antioxidant activity, attenuation of SASP, promotion of NAD^+^ production, and mitigation of mitochondrial dysfunction. The results suggest that GOPs exert their beneficial effects through multiple mechanisms. Firstly, their antioxidant activity helps counteract oxidative stress, which is a major contributor to cellular damage and aging. Secondly, GOPs exhibit anti-inflammatory effects by downregulating NF-κB, a key regulator of the inflammatory response. This anti-inflammatory action contributes to the preservation of cellular function and integrity. Thirdly, GOPs promote mitochondrial biogenesis through the NAD^+^/SIRT1/PGC-1α pathway, which enhances mitochondrial function and energy production, especially in HUVECs. Lastly, our results, regarding PC12 cells, indicate that GOPs may exert their protective effects on neuronal cells by modulating the mTOR/ULK1/Beclin1/LC3B pathway, leading to a reduction in autophagic cell death. Overall, the findings from our study suggest that GOPs possess a range of beneficial properties that can positively influence aging processes. By combating cellular damage (senescence and death), oxidative stress, and inflammation, while protecting mitochondria, GOPs have the potential to extend both the lifespan and health span.

This study has a limitation wherein the oxidative stress-induced damage model may not entirely mimic the natural cellular senescence process. Hence, additional investigations involving primary cell cultures and in vivo experiments are imperative to yield more conclusive evidence. Moreover, further research is warranted to ascertain the ideal dosage of GOPs for cellular exposure, and to comprehensively comprehend the impact of GOPs on cell metabolism and the associated physiological functions. It is crucial to emphasize that more extensive studies are required to unveil the exact role of GOPs in extending both the lifespan and health span.

## 4. Materials and Methods

### 4.1. COPs

The GOPs were provided by Jilin TAIGU Biological Engineering Co., Ltd. (Jilin, China). These oligopeptides were obtained from the roots of *Panax ginseng C.A. Meyer* through enzymatic hydrolysis, as previously described [19]. The molecular weight of GOPs ranging from 100 to 1000 Dalton accounted for 95.42% of GOPs, while the free amino acids amounted to 3.94%. Table 1 provides more detailed information.

### 4.2. Cell Culture and Treatments

The HUVECs and PC-12 cells used in this study were sourced from the American Type Culture Collection (ATCC, Manassas, VA, USA). They were incubated in Dulbecco’s modification of Eagle medium (DMEM) (GIBCO, Grand Island, NY, USA) with 10% fetal bovine serum (Gibco, Grand Island, NY, USA) and 1% antibiotic–antimitotic (Coolaber, Beijing, China) at 37 °C in 5% CO_2_.

For the oxidative-induced damaged model, HUVECs and PC-12 were treated with different concentrations of H_2_O_2_, ranging from 50 to 800 µM for 4 h, and were then maintained in a growth medium for 24 h. The effective intervention dose of H_2_O_2_ for HUVECs was evaluated by assessing the cell viability and the relative protein expression of p16^INK4A^ and p21^Waf1/Cip1^. For PC-12, the cell viability was tested after 24 h and the optimal H_2_O_2_ concentration was chosen for the subsequent experiment.

The current research involved 5 groups: (1) the control group: cells were cultured in a growth medium; (2) the H_2_O_2_-treated GOP-free group: growth medium with 200 µM of H_2_O_2_ was used for 4 h, and then, the cells were maintained in a growth medium without H_2_O_2_ for 24 h; (3) 3 doses of GOP groups: cells were supplemented with a growth medium containing 200 µM of H_2_O_2_ and 25, 50, and 100 µg/mL of GOPs for 4 h, then incubated with a H_2_O_2_-free growth medium with different concentrations of GOPs. After exposure to GOPs, the cells were harvested for further investigation.

### 4.3. Cell Viability Assay

For the cell viability assay, 100 μL/well cells (about 1 × 10^4^) were cultured in 96-well plates, and treated according to the experimental protocol: 10 μL of a CCK-8 assay solution (KeyGEN, Jiangsu, China) was added to each well, and incubated for 1–4 h at 37 °C. The absorbance of each well was detected using a microplate reader (BMG FLUOstar Omega, Offenburg, Germany) at a 450 nm wavelength.

### 4.4. Flow Cytometry

For flow cytometry, 2 mL/well cells (about 2 × 10^5^) were incubated in 6-well plates and treated according to the protocol. The cells were digested and washed twice with phosphate-buffered saline (PBS) and then fixed with 75% ethanol overnight at 4 °C. Then, the cells were washed three times with PBS, and incubated with propidium staining and RNase A (Beyotime, Shanghai, China) at 37 °C for 30 min. Then, the cell cycle was analyzed using a flow cytometer (Beckman Coulter, Brea, CA, USA). The cells were harvested and washed once with PBS and incubated for 20 min at 37 °C with 10 μM of 2,7-dichlorofluorescein diacetate (Beyotime, Shanghai, China). After being washed with PBS three times, the production of reactive oxygen species (ROS) was analyzed using a flow cytometer. The cells were collected and stained with 500 µL of 1 × JC-1 dye solution (Beyotime, Shanghai, China) at 37 °C for 20 min in the dark. Then, the cells were washed twice and resuspended using a 1 × JC-1 staining buffer, and the mitochondrial membrane potential (∆Ψm) was analyzed using flow cytometry. The cells were digested and washed once with PBS and then resuspended in a PI/Annexin-V solution (KeyGEN, Jiangsu, China). Finally, the apoptosis rate was analyzed using a flow cytometer.

### 4.5. Biochemical Analysis

For biochemical analysis, 2 mL/well cells (about 2 × 10^5^) were cultured in 6-well plates and treated according to the experimental protocol. The supernatant was collected to measure the levels of malondialdehyde (MDA) (Nanjing Jiancheng, Nanjing, China), glutathione peroxidase (GSH-Px) (Nanjing Jiancheng, Nanjing, China), and superoxide dismutase (SOD) (Nanjing Jiancheng, Nanjing, China) activities, as well as NAD^+^/NADH (Beyotime, Shanghai, China) and telomerase (TE) activities (FEIYA, Jiangsu, China). The secretion of interleukin-6 (IL-6) (Invitrogen, Waltham, MA, USA), IL-1β (Invitrogen, Waltham, MA, USA), matrix metalloproteinase-3 (MMP-3) (Multisciences, Hangzhou, China), and intercellular cell adhesion molecule-1 (ICAM-1) (Multisciences, Hangzhou, China) were also measured, according to the protocol of commercial kits.

### 4.6. Western Blot Analysis

For Western blot analysis, 2 mL/well cells (about 2 × 10^5^) were cultured in 6-well plates and treated according to the experimental protocol. Then, the cells were digested and washed twice with PBS and maintained in RIPA Lysis Buffer (Biosharp, HeFei, China) containing 1 mM of phenylmethanesulfonyl fluoride. Cell protein was extracted through centrifugation at 14,000× *g* for 15 min at 4 °C, and the protein content was evaluated using a BCA protein assay kit (Thermo Scientific, Waltham, MA, USA). An equal quantity of protein (80–150 ug) was separated using 10–20% SDS-PAGE gel and subsequently transferred onto PVDF membranes using varying electric currents based on the size of the protein molecules. The membranes were blocked with a solution of 5% nonfat milk dissolved with Tris-buffered saline containing 0.05% Tween-20 (TBST) at room temperature for 2 h. Protein expression was detected using a primary antibody p16^INK4A^ (1:1000, CST, Danvers, MA, USA), γ-H2A.X (1:1000, Abcam, Cambridge, MA, USA), p-AMPKα (1:1000, CST, Danvers, MA, USA), AMPKα (1:1000, CST, Danvers, MA, USA), PGC-1α (1:2000, Abcam, Cambridge, MA, USA), SIRT1 (1:1000, CST, Danvers, MA, USA), mTOR (1:1000, Abcam, Cambridge, MA, USA), p-mTOR (1:1000, Abcam, Cambridge, MA, USA), ULK1 (1:1000, Abcam, Cambridge, MA, USA), LC-3B (1:1000, Abcam, Cambridge, MA, USA), Beclin1 (1:1000, Abcam, Cambridge, MA, USA), and β-actin (1:5000, Abcam, Cambridge, MA, USA), and horseradish peroxidase-conjugated antirabbit secondary antibodies (1:10,000, Abcam, Cambridge, MA, USA) were utilized. Quantitative analysis of the Western blot was conducted using Image-Pro Plus (Media Cybernetics, Rockville, MD, USA).

### 4.7. Statistical Analysis

The SPSS software version 24 (SPSS Inc., Chicago, IL, USA) was utilized for statistical analysis. Data are expressed as the mean ± standard deviation (SD) and analyzed using a one-way analysis of variance (ANOVA) test. To analyze the difference in parametric samples among different groups, multiple comparisons of the least significant difference (equal variances assumed) or Dunnett’s T3 test (equal variances not assumed) was utilized. Statistically significant difference was indicated by *p* < 0.05.

## 5. Conclusions

In summary, the emerging understanding of the beneficial effects of GOPs on cellular processes, including their impact on oxidative stress, inflammation, and mitochondrial function, holds great promise for their potential applications in diverse research fields. It is important to note that while existing evidence is promising, further research is needed to fully explore the potential applications of GOPs in these fields. Additional studies, including in vivo and clinical trials, will be valuable in elucidating the specific mechanisms of action, optimal dosage, and potential side effects of GOPs. Nonetheless, the current understanding of the bioactivity of GOPs provides a solid foundation for expanding research into their wide-ranging applications.

## Figures and Tables

**Table 1:** The amino acid composition of ginseng oligopeptides (GOPs).

Amino Acid	Content (g/100 g)	Amino Acid	Content (g/100 g)
Aspartic Acid	0.19	Cystine	0.01
Glutamic Acid	0.12	Valine	0.06
Serine	0.02	Methionine	0.02
Histidine	0.06	Phenylalanine	0.09
Glycine	0.02	Isoleucine	0.04
Threonine	0.05	Leucine	0.08
Arginine	2.26	Lysine	0.06
Alanine	0.13	Proline	0.65
Tyrosine	0.09		

## Data Availability

The data presented in this study are available on request from the corresponding author.
